# Epicardial Adipose Tissue Is Associated with Plaque Burden and Composition and Provides Incremental Value for the Prediction of Cardiac Outcome. A Clinical Cardiac Computed Tomography Angiography Study

**DOI:** 10.1371/journal.pone.0155120

**Published:** 2016-05-17

**Authors:** Gitsios Gitsioudis, Christina Schmahl, Anna Missiou, Andreas Voss, Alena Schüssler, Hassan Abdel-Aty, Sebastian J. Buss, Dirk Mueller, Mani Vembar, Mark Bryant, Hans-Ulrich Kauczor, Evangelos Giannitsis, Hugo A. Katus, Grigorios Korosoglou

**Affiliations:** 1 University of Heidelberg, Department of Cardiology, Heidelberg, Germany; 2 University of Heidelberg, Institute of Psychology, Heidelberg, Germany; 3 CT Clinical Science, Philips Healthcare GmbH, Hamburg, Germany; 4 CT Clinical Science, Philips Healthcare, Cleveland, Ohio, United States of America; 5 University of Heidelberg, Department of Diagnostic and Interventional Radiology, Heidelberg, Germany; Medical Faculty, Ludwig Maximilians University Munich, GERMANY

## Abstract

**Objectives:**

We sought to investigate the association of epicardial adipose tissue (eCAT) volume with plaque burden, circulating biomarkers and cardiac outcomes in patients with intermediate risk for coronary artery disease (CAD).

**Methods and Results:**

177 consecutive outpatients at intermediate risk for CAD and completed biomarker analysis including high-sensitive Troponin T (hs-TnT) and hs-CRP underwent 256-slice cardiac computed tomography angiography (CCTA) between June 2008 and October 2011. Patients with lumen narrowing ≥50% exhibited significantly higher eCAT volume than patients without any CAD or lumen narrowing <50% (median (interquartile range, IQR): 108 (73–167) cm^3^ vs. 119 (82–196) cm^3^, p = 0.04). Multivariate regression analysis demonstrated an independent association eCAT volume with plaque burden by number of lesions (R^2^ = 0.22, r_partial_ = 0.29, p = 0.026) and CAD severity by lumen narrowing (R^2^ = 0.22, r_partial_ = 0.23, p = 0.038) after adjustment for age, diabetes mellitus, hyperlidipemia, body-mass-index (BMI), hs-CRP and hs-TnT. Univariate Cox proportional hazards regression analysis identified a significant association for both increased eCAT volume and maximal lumen narrowing with all cardiac events. Multivariate Cox proportional hazards regression analysis revealed an independent association of increased eCAT volume with all cardiac events after adjustment for age, >3 risk factors, presence of CAD, hs-CRP and hs-TnT.

**Conclusion:**

Epicardial adipose tissue volume is independently associated with plaque burden and maximum luminal narrowing by CCTA and may serve as an independent predictor for cardiac outcomes in patients at intermediate risk for CAD.

## Introduction

Epicardial adipose tissue (eCAT) belongs to the endocrine active assemblage of visceral body fat with paracrine impact on the initiation and progression of coronary artery disease (CAD) [1–4].

Previous large cohort studies demonstrated that eCAT volume is associated with atherogenic risk factors, the presence of CAD and plaque burden [3, 5–9]. This observation is supported by the evidence of metabolic activity of eCAT as a source of several proatherogenic mediators, accompanied by paracrine or vasocrine mechanisms [10]. Furthermore, growing body of evidence suggests that elevated eCAT volume is independently associated with increased incidence of future myocardial infarction [11–13]. High-sensitive Troponin T (hs-TnT), on the other hand, is a sensitive biomarker of myocardial injury associated with high-risk coronary lesions and plaque burden and provides incremental value for the prediction of cardiac outcome in patients with both presumably stable CAD and preserved systolic left ventricular function [14–17]. Hs-CRP is a surrogate of inflammation associated with CAD and cardiac outcome [15, 17–19]. However, little evidence exists on the impact of eCAT volume on both cardiac troponins and hs-CRP, respectively. Cardiac computed tomography angiography (CCTA) enables for a simultaneous quantitative assessment of atherosclerotic plaque and eCAT volume [17, 20–22]. Recently, a strong association of eCAT volume with non-calcified plaque composition was reported [5, 8, 9]. However, to the best of our knowledge, the association of eCAT volume and quantitative plaque composition with biomarkers like hs-TnT and hs-CRP has not been reported so far.

Herein, we therefore assessed the role of eCAT volume for coronary plaque burden by CCTA, its association with established biomarkers of myocardial injury (hs-TnT) and inflammation (hs-CRP), and investigated its prognostic value in presumably stable CAD patients.

## Methods

### Study population

**Fig 1:**
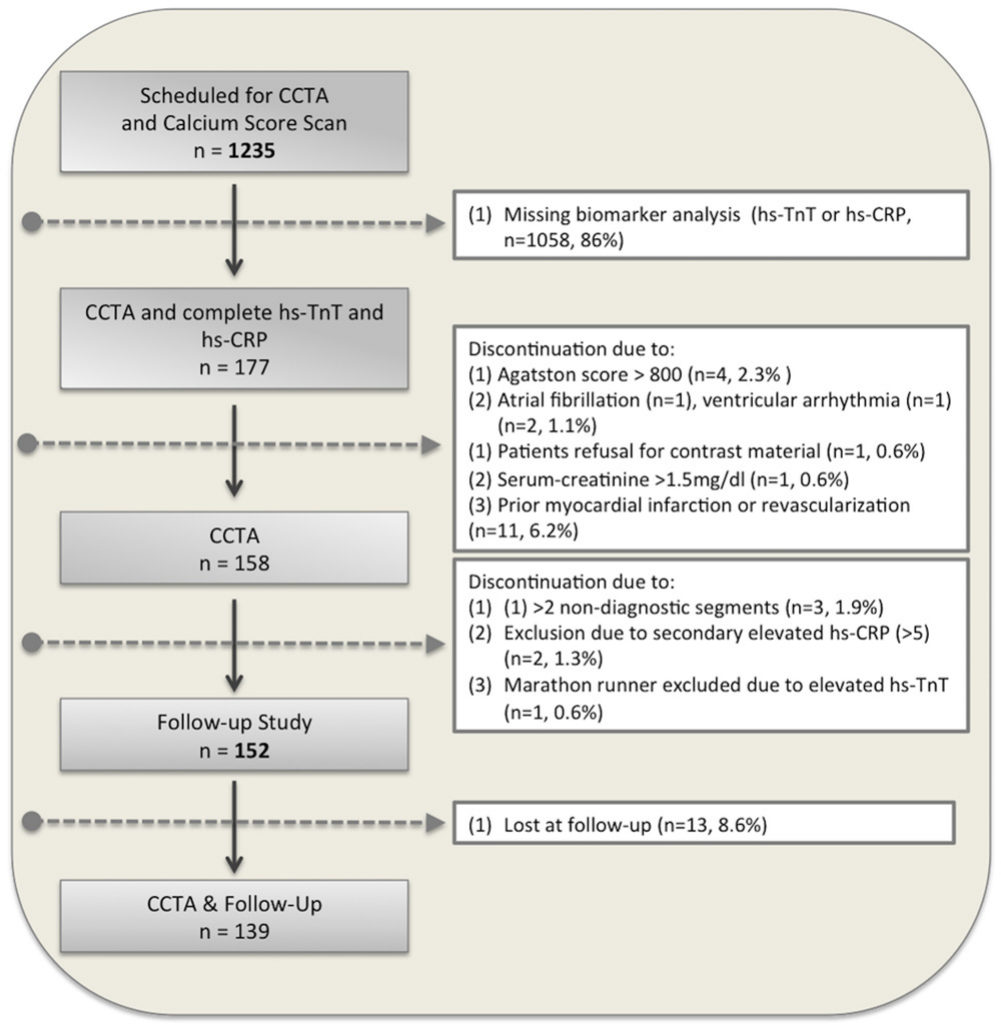
Flow diagram of patient enrolment.

A total of 1235 consecutive outpatients were scheduled for cardiac computed tomography angiography (CCTA) due to suspected or known coronary artery disease (CAD) between June 2008 and October 2011. CCTA was performed for clinical reasons according to the current guidelines [23]. All imaging was performed with a 256– detector row CT scanner (iCT; Philips Medical Systems, Best, the Netherlands) with a 2x128x0.625 mm detector configuration, as described previously [24]. Inclusion and exclusion criteria are provided online (S1 Appendix). The assessment of demographic and clinical characteristics is described online (S1 Appendix) and summarized in Table 1. We prospectively included 177 (14%) patients in our observational longitudinal single-center study who had a completed biomarker analysis for hs-TnT and hs-CRP (Fig 1). 25 patients were excluded due to the presence of one or more exclusion criteria, as listed online (S1 Appendix, Fig 1). An additional 13 patients were lost at follow-up, so that our final study population comprised 152 patients (87 men, mean age 64±10 years), and 139 patients with completed follow-up (Fig 1). Our study complied with the Declaration of Helsinki, was approved by our local ethics committee of the University of Heidelberg (S317/2008) and all patients gave written informed consent.

**Table 1:** Demographic, laboratory and cardiac computed tomography angiography (CCTA) findings in patients with and without cardiac events.

Variable	All Patients (n = 152)	Patients w/o CAD or Luminal Narrowing <50% (n = 104)	Patients with Luminal Narrowing ≥50% (n = 48)	P value
	**Clinical data**			
Age, years	64±10	63±9	67±11	NS
1. Advanced age > 65yrs.	74 (49%)	42(40%)	32(67%)	NS
2. Male gender, %	87 (57%)	54(52%)	33 (69%)	NS
3. Arterial hypertension	121 (80%)	80(77%)	41(85%)	**0.03**
4. Hyperlipidemia	87 (57%)	56(54%)	31(65%)	NS
5. Smoking	64 (42%)	44(42%)	20(42%)	NS
6. Diabetes mellitus	14 (9%)	8(8%)	6 (13%)	NS
7. Family history of CAD	70 (46%)	49(47%)	21(44%)	NS
Sum of risk factors (0–7)	2.5±1.2	2.4±1.1	3.0±1.2	**0.003**
Body mass index, kg/m²	27.3±4.8	26.6±4.2	28.6±5.7	**0.02**
Duke Clinical Score, %	60±29	58±28	66±29	NS
	**Laboratory data & biomarkers**			
Serum creatine, mg/dl	0.9±0.2	0.9±0.2	1.0±0.3	**0.02**
Serum urea, mg/dl	36.5±10.4	35.3±9.5	40.0±11.5	**0.01**
GFR-MDRD, ml/min/1.73m^2^	79.9±19.8	81.2±19.5	77.0±21.0	NS
Hs-CRP, mg/l	2.3±2.4	2.1±2.1	2,7±2.3	NS
Hs-TnT, pg/ml	10.7±6.1	10.3±5.1	11.6±8.0	NS
Total Cholesterol, mg/dl	200.1±43.6	204±44	189.0±42.2	NS
LDL-Cholesterol, mg/dl	116.2±36.7	119±37.0	106.2±36.3	NS
HDL-Cholesterol, mg/dl	55.2±16.7	55±17	55.3±18.2	NS
Triglycerides, mg/dl	140.6±104.6	143±117	136.7±74.3	NS
	**Cardiac computed tomography data**			
Calcium score, Agatston Units	52 (0–320)	9 (0–174)	247 (39–522)	**<0.001**
Number of plaques per patient	1.0 (0–3.0)	0.0 (0–2.0)	2.5 (1.0–4.9)	**<0.001**
Total plaque volume, mm³	9.2 (0–83.0)	0.0 (0–49.5)	67.5 (9.2–178.1)	**<0.001**
Non-calc. plaque volume, mm³	0.0 (0–28.2)	0.0 (0–14.6)	13.2 (0–83.9)	**<0.001**
Maximal lumen narrowing, %	38.0 (0–61.7)	0.0 (0–42.3)	54.5 (26.4–76.0)	**<0.001**
Positive remodeling, %	36(24%)	11 (10%)	25(56%)	**<0.0001**
ECAT volume, cm^3^	116 (80–174)	108 (73–167)	119.0 (82–196)	**0.04**
	**Cardiac medications**			
ß-blockers, %	74(49%)	49(46%)	25(56%)	NS
ACE inhibitors/AT II blockers, %	36(24%)	26 (25%)	10 (22%)	NS
Aspirin or Clopidogrel, %	79 (52%)	46 (44%)	33(69%)	**0.004**
Diuretics, %	29 (19%)	19(23%)	10(27%)	NS
Statins, %	69(45%)	36(34%)	33(73%)	**<0.001**

CCTA-based data are presented as median (interquartile range), all other data are presented mean±SD or as proportions. Hs-TnT indicate high sensitive troponin T; hs-CRP, high-sensitive C-reactive protein; eCAT, epicardial adipose tissue; ACE, angiotensin-converting enzyme; AT, angiotensin; GFR-MDRD, Glomerular Filtration Rate estimated by the Modification of Diet in Renal Disease method.

### Patient preparation and CCTA imaging protocols

Patient preparation included the intravenous administration of 2.5–30.0 mg metoprolol (Lopresor^®^, Novartis, Pharma GmbH) if baseline heart rate was more than 60 beats per minute. All patients received 0.8 mg of sublingual glyceryl nitrate 5 minutes before the CT scan. During a single breath-hold, CCTA was performed with 65–80 ml (injection rate 6 ml/s) of nonionic contrast agent (Ultravist^®^ 370, Bayer Schering Pharma) followed by 30 ml (injection rate 5 ml/s) of saline that was administrated using an antecubital line. Imaging parameters were used as previously described [25] with n = 112 (71%) undergoing prospectively ECG triggered and n = 46 (29%) undergoing retrospectively ECG gated scans.

### Quantification of epicardial adipose tissue (eCAT)

According to previous reports we performed all measurements with dedicated software (Extended Brilliance Workspace 4.0, Philips Healthcare). First, we identified the following anatomic boundaries for measurement of total eCAT volume: (i) upper boundaries: pulmonary artery bifurcation, the mid left atrium, and the aortic root, (ii) lower boundaries: the diaphragm and the left ventricular apex. Second, we defined the lower density threshold as -190 HU and the upper density threshold as -30 HU for subsequent 3D-segmentation [26].

### Computer assisted evaluation of plaque volume, composition and luminal narrowing

The methods used for evaluation of diagnostic image quality, visual plaque evaluation and quantitative assessment of Agatston score, luminal narrowing, coronary plaque volume and composition using the dedicated software (Extended Brilliance Workspace 4.0, Philips Medical Systems) have been previously established and described [17, 20, 27] and is provided online (S1 Appendix). Coronary CT angiograms and Agatston score were analyzed independently by two experienced readers (G.G. & G.K.) both with >5 years of experience in CCTA equivalent to the clinical competence statement training level 3 of the American College of Cardiology Foundation/American Heart Association (AHA) [28]. The per-patient fraction of non-calcified (FR_non-calc.)_ or calcified (FR_calc._) plaque content in patients with at least one coronary plaque was calculated as follows:

$$\text{F}{\text{R}}_{\left(\text{non}-\right)\text{calcified}}=\text{ Total }\left(\text{non}-\right)\text{calcified plaque volume }/\text{ Total plaque volume}$$ (1.1)

### Agatston score

For the assessment of coronary calcification prospective ECG-gated non-contrast scans were performed at 75% of the cardiac cycle, and using 120 kV tube voltage and 364 mA tube current, and resultant images with a 3 mm slice thickness were used for the calculation of the Agatston score.

### Follow-up and study endpoints

Personnel who were unaware of the CCTA results contacted each subject or an immediate family member. The date of this contact was used for the calculation of the follow-up time duration. A standardized questionnaire was used to collect outcome data determined from patient interviews at the outpatient clinic or by telephone interviews. Reported clinical events were confirmed by review of the corresponding medical records in our electronic Hospital Information System, and contact with the general practitioner, referring cardiologist, or the treating hospital. The pre-specific endpoints of this study were cardiac death (sudden death due to arrhythmia, fatal myocardial infarction (MI) or intractable heart failure) and nonfatal MI. Further cardiac events included the occurrence of clinically indicated revascularization procedures by percutaneous coronary intervention (PCI) or coronary artery bypass graft surgery (CABG). MI was defined according to the European Society of Cardiology/American College of Cardiology Universal MI Definitions Committee, and for unstable angina, the Braunwald classification was used [29, 30]. Since CCTA results may have triggered revascularization procedures, thereby altering the subsequent event rate, ‘early’ revascularization within 90 days of CCTA was not considered, and patients were censored at the time of early revascularization (n = 6).

### Biomarkers

Blood samples were drawn from all patients before the CCTA scan. Analysis included both biomarkers hs-TnT and hs-CRP and routine laboratory parameter measurements. A detailed description of biomarker analysis is available online (S1 Appendix).

### Statistical analysis

Statistical analyses were performed with use of MedCalc software (MedCalc 15.11.0, Ostend, Belgium). Categorical variables are presented as proportions (%). Continuous variables as mean ± standard deviation (SD) or median and interquartile range (IQR), as appropriate. Normality of data distribution was evaluated using Kolmogorov-Smirnov test. Since part of the continuous variables in Tables 1 and 2 showed a non-normally distribution, all CCTA-derived values are uniformly presented as median and IQR. ECAT was normally distributed. Inter-group comparisons were made using either the unpaired *t* test for continuous variables, the Mann-Whitney U test for ordinal variables and the Fisher’s exact test for nominal variables. All tests were 2-tailed. ECAT tertiles were defined as follow: 1^st^ tertile: <97 cm^3^; 2^nd^ tertile: 97 to 142 cm^3^; 3^rd^ tertile: >142 cm^3^. For survival analysis the cut-off value of ≥75th percentile (≥162.2 cm^3^) was defined for elevated eCAT volume. For hs-TnT and hs-CRP clinically established cut-off values of 14 pg/ml and 5 mg/l were used, respectively [15, 17]. For categorization of normal and high plaque volume we used a cut-off value of 19.6 mm^3^ [17]. To account for non-normally distributed CCTA-based variables (for example total plaque volume) we performed Spearman’s correlation analysis. For all other correlation analyses we calculated the Pearson correlation coefficient r, with p value. Multiple linear regression models were calculated to analyze the relationship between total plaque volume, calcium score, fraction of non-calcified plaque volume and the traditional risk factors, biomarkers and eCAT volume. Results are reported as the coefficient of determination R^2^ as the proportion of the variation in the dependent variable (e.g. total plaque volume) and the partial correlation coefficient r_partial_ as the coefficient of correlation of the tested variable with the dependent variable, adjusted for the effect of the other variables in the model. For survival analysis, Kaplan-Meier curves were generated to estimate the distribution of cardiac events as a function of the follow-up duration, depending on the presence or absence of elevated eCAT volume. Cox proportional-hazards univariate and multivariable regression analysis with Bonferroni adjustment for multiple comparisons was performed to identify predictors of all cardiac events (MI and cardiac death and late revascularization). Baseline variables that were considered clinically relevant (>3 risk factors for CAD, BMI, hs-CRP and hs-TnT) or that showed a univariate relationship with outcome were entered into the analysis. Results are presented as Hazard Ratios (HR) with the 95% confidence interval (95%CI) and the b-coefficient for multivariable analyses. In addition, we calculated the category-less net reclassification improvement (NRI) by using the “survIDINRI” software package (Revolution Analytics, Mountain View, California, USA). For reproducibility of eCAT volumes, we used the intra-class correlation coefficient (ICC) for intra-observer and inter-observer agreement and paired t-test for determining the significance of the mean absolute differences for repeated analysis of 40 randomly selected CCTA cases. The readings were separated by 8 weeks to minimize recall bias. A p value <0.05 was considered statistically significant.

**Table 2:** Distribution of clinical, laboratory and computed tomography angiography (CCTA) findings by tertile analysis of epicardial adipose tissue (eCAT) volume.

Variable	1^st^ eCAT tertile (n = 50)	2^nd^ eCAT tertile (n = 51)	3^rd^ eCAT tertile (n = 51)	P value
	**Clinical data**			
Age, years	63±10	63±9	66±8	ns
1. Advanced age > 65yrs.	20 (40%)	23 (46%)	29 (57%)	ns
2. Male gender, %	16 (33%)	33(64%)	40 (79%)	**<0.001**
3. Arterial hypertension	31 (62%)	42 (83%)	47 (91%)	**≤0.002**
4. Hyperlipidemia	26(51%)	31 (61%)	33 (65%)	ns
5. Smoking	18 (36%)	18 (35%)	28 (54%)	ns
6. Diabetes mellitus	3 (7%)	6 (11%)	6 (11%)	ns
7. Family history of CAD	24 (49%)	29 (57%)	19 (37%)	ns
Sum of risk factors (0–7)	2.2±1.2	2.6±1.1	2.9±1.2	**<0.05**
Body mass index, kg/m²	25.2±4.1	27.6±4.1	29.5±5.2	**<0.05**
Duke Clinical Score, %	51±30	61±27	70±26	**<0.05**
	**Laboratory data & biomarkers**			
Serum creatine, mg/dl	0.8±0.2	0.9±0.2	1.0±0.3	**<0.05**
Serum urea, mg/dl	35.0±8.7	33.8±8.4	41.2±12.4	**<0.05**
GFR-MDRD, ml/min/1.73m^2^	82.2±18.5	82.8±17.7	75.0±21.7	ns
Hs-CRP, mg/l	2.0±2.5	2.0±1.7	2.9±2.4	ns
Hs-TnT, pg/ml	10.7±6.1	10.6±4.8	11.6±7.8	ns
Total Cholesterol, mg/dl	212.3±42.8	193.7±50.0	194.6±38.5	ns
LDL-Cholesterol, mg/dl	117.2±40.7	118.2±35.7	115.3±34.7	ns
HDL-Cholesterol, mg/dl	65.7±16.1	48.3±10.5	46.8±14.0	**<0.05**
Triglycerides, mg/dl	104.0±38.6	116.8±44.3	210.7±160.1	**<0.05**
	**Cardiac computed tomography data**			
Calcium score, Agatston Units	9 (0–269)	84 (0–361)	117 (0–486)	**0.02**
Number of plaques per patient	0.0 (0.0–2.0)	2.0 (0.0–4.0)	2.0 (0.0–5.5)	**<0.001**
Total plaque volume, mm³	0.0 (0.0–62.1)	18.4 (0.0–140.3)	36.9 (0.0–165.3)	**0.004**
Non-calc. plaque volume, mm³	0.0 (0.0–8.3)	2.2 (0–73.1)	2.2 (0–72.2)	**0.009**
Maximal lumen narrowing, %	0.0 (0.0–62.0)	39.5 (0.0–66.8)	47.5 (0.0–69.0)	**0.004**
Lumen narrowing >70%, %	2 (5%)	5 (9%)	7 (13%)	ns
Positive remodeling, %	12 (24%)	11 (22%)	16 (30%)	ns
ECAT volume, cm^3^	77 (56–91)	113 (101–135)	177 (155–228)	**<0.001**

CCTA-based data are presented as median (interquartile range), all other data are presented mean±SD or as proportions. Hs-TnT indicates high sensitive troponin T; hs-CRP, C-reactive protein; GFR-MDRD, Glomerular Filtration Rate estimated by the Modification of Diet in Renal Disease method.

## Results

### Baseline characteristics and CCTA results

Patients with obstructive CAD (n = 48) (lumen narrowing 50–70% (n = 39) or >70% (n = 9), respectively) presented higher total number of atherogenic risk factors and higher BMI compared to those without any CAD or CAD with lumen narrowing <50% (n = 104, Table 1). Overall, the Duke clinical score was 60±29% indicating an intermediate likelihood for CAD. Diagnostic image quality was present in 2,244 of 2,280 (98.4%) coronary segments.

### Associations between eCAT with traditional risk factors and biomarkers

**Fig 2:**
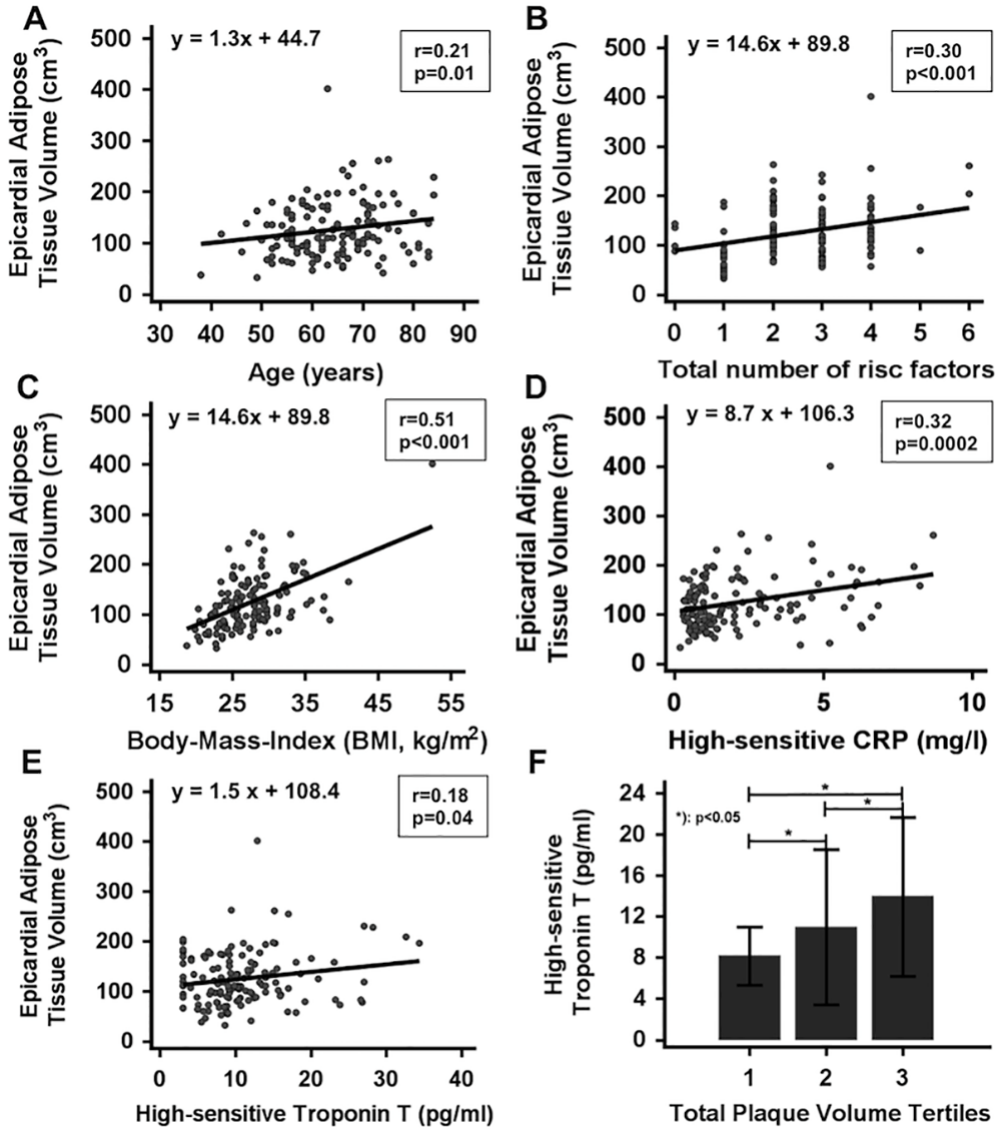
Correlation analysis for eCAT (epicardial adipose tissue) volume with age (**A**), total number of atherogenic risk factors (**B**), BMI (**C**), hs-CRP (**D**) and hs-TnT (**E**). Association of small increases of hs-TnT (mean±SD) with tertiles of total plaque volume (**F**). BMI, body-mass-index; hs-CRP, high-sensitive C-reactive protein; hs-TnT, high-sensitive Troponin T; SD, standard deviation.

Univariate regression analysis demonstrated an association of eCAT volume with age, total number of atherogenic risk factors, BMI and the biomarkers hs-CRP and hs-TnT (Fig 2A–2E). In addition, significant correlations were observed between hs-TnT and total plaque volume (Fig 2F) and between eCAT volume and serum lipid levels (S1 Fig).

### Associations between eCAT and plaque composition

**Fig 3:**
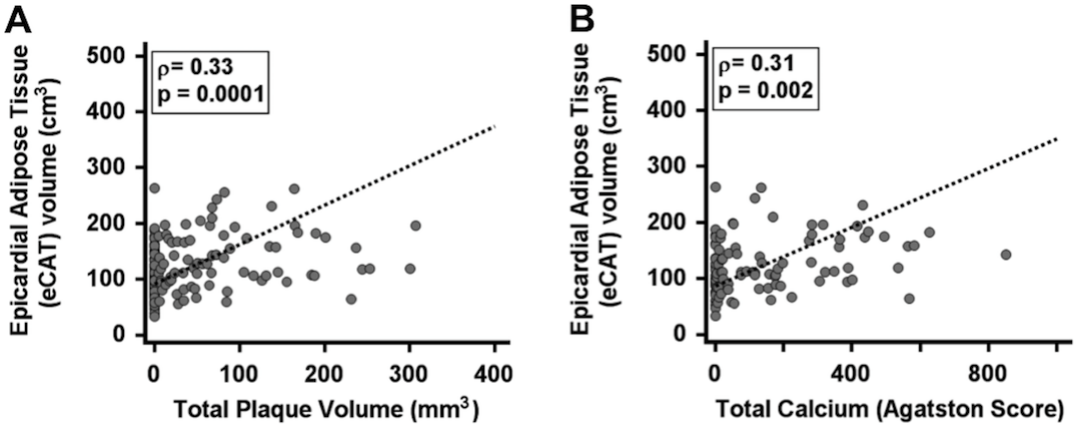
Spearman’s correlation analysis reveals a significant correlation of epicardial adipose tissue (eCAT) volume with total plaque volume (A) and total calcium score by Agatston (B).

Overall, 184 coronary artery plaques (101 calcified, 52 mixed and 31 non-calcified) were detected and quantitatively analyzed. Spearman’s correlation analysis demonstrated a significant correlation of eCAT volume with total plaque volume (ρ = 0.33, p = 0.0001, 95%CI 0.17 to 0.47, Fig 3A), calcium score (ρ = 0.31, p = 0.002, 95%CI 0.12 to 0.48, Fig 3B) and fraction of non-calcified plaque volume (ρ = 0.26, p = 0.011, 95%CI 0.06 to 0.43). Multiple linear regression analysis revealed an independent association a of eCAT volume with total plaque volume (R^2^ = 0.11, r_partial_ = 0.22, p = 0.020), with calcium score (R^2^ = 0.32, r_partial_ = 0.26, p = 0.010) and with fraction of non-calcified plaque volume (R^2^ = 0.23, r_partial_ = 0.27, p = 0.014) after adjustment for age, diabetes mellitus, hyperlidipemia, BMI, hs-CRP and hs-TnT.

### ECAT and CAD severity

**Fig 4:**
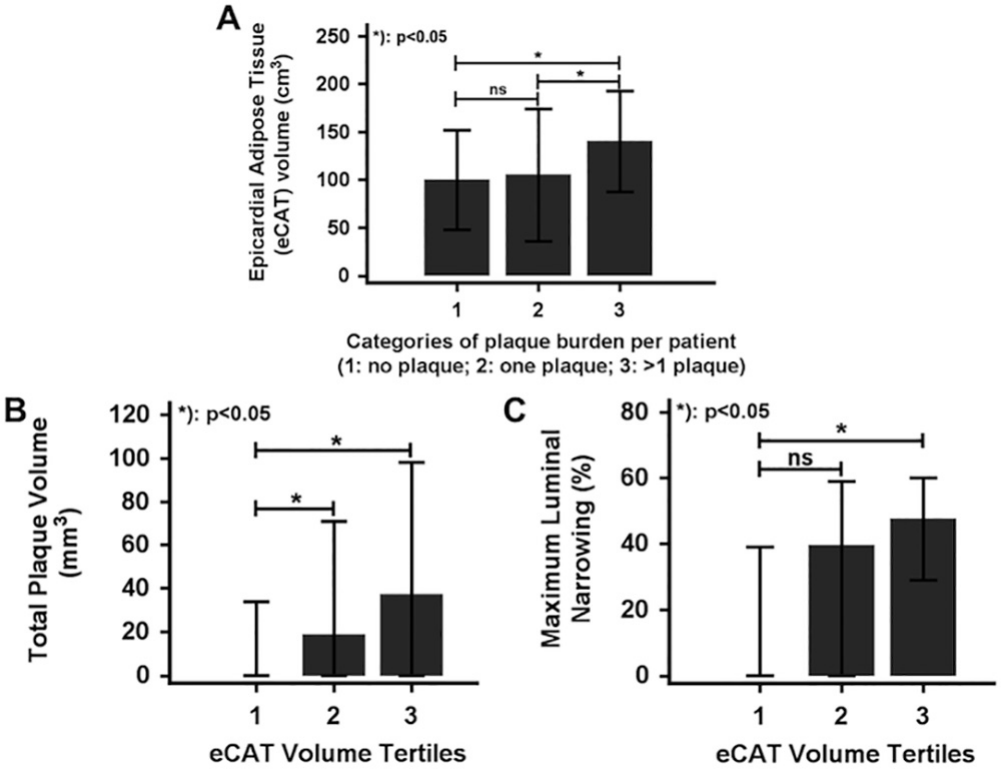
**A**: Patients with >1 plaque (group 3, n = 71) exhibit significantly higher epicardial adipose tissue (eCAT) volumes compared to patients without any plaque (group 1, n = 68) or only one plaque (group 2, n = 13). **B/C**: Analysis per tertiles reveals an increase of total plaque volume (**A**) and maximum coronary luminal narrowing (**B**) for higher eCAT (epicardial adipose tissue) volumes. (bars: median ± interquartile range, IQR)

Patients with >1 plaque (n = 71, eCAT volume: 140±53 cm^3^,) exhibited a significantly increased eCAT volume compared to patients without any plaque (n = 68, eCAT volume: 99±52 cm^3^) and those with one plaque (n = 13, eCAT volume: 105±69 cm^3^), respectively (p<0.05 for both, Fig 4A). Analysis by tertiles identified a significant association of eCAT volume with total plaque volume and maximum lumen narrowing (Table 2, Fig 4B and 4C). In addition, eCAT volume was independently associated with presence of CAD (any plaque or luminal narrowing, R^2^ = 0.11, r_partial_ = 0.21, p = 0.026), plaque burden (by number of lesions: R^2^ = 0.22, r_partial_ = 0.29, p = 0.006) and CAD severity (by maximum lumen narrowing: R^2^ = 0.22, r_partial_ = 0.23, p = 0.038) after adjustment for age, diabetes mellitus, hyperlidipemia, BMI, hs-CRP and hs-TnT.

### ECAT and cardiac outcomes

During a 3.2±1.1 year follow-up period (median 3.3, range 0.5–6.3) 10 hard cardiac events occurred (5 non-fatal myocardial infarctions and 5 cardiac deaths), while 6 patients underwent late revascularizations.

**Fig 5:**
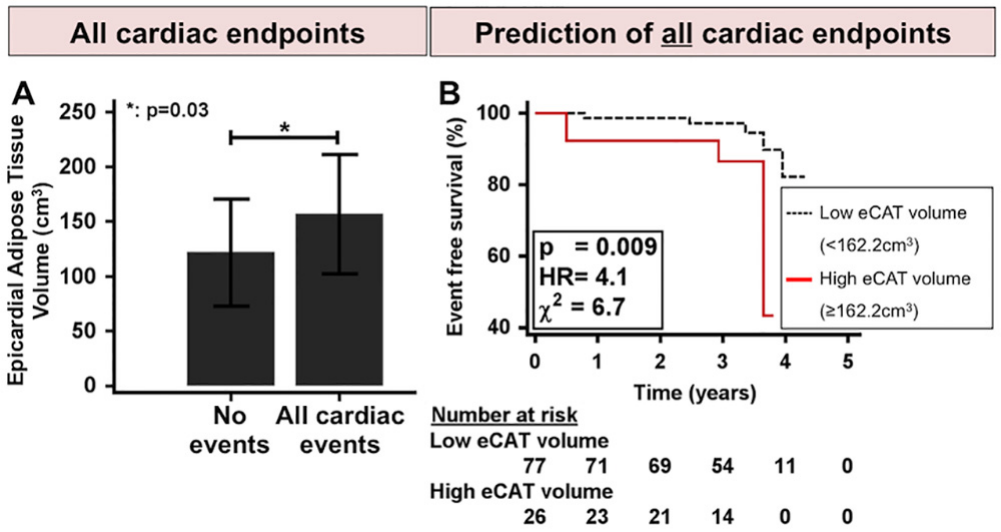
Survival analysis. **A**: Event group patients exhibit higher eCAT (epicardial adipose tissue) volumes than non-event patients. **B**: Kaplan-Meier survival analysis reveals poorer outcome for patients with elevated eCAT volume (≥162.2 cm^3^) compared to patients with low eCAT volume.

Patients with cardiac events exhibited higher eCAT volumes than patients without cardiac events (156.6±58.2 cm^3^ vs. 121.5±49.1 cm^3^, p = 0.03) (Fig 5A). Using univariate Cox proportional hazards regression analysis, significant associations were observed for both increased eCAT volume and maximal lumen narrowing in CCTA with all cardiac events (Table 3). Using multivariate Cox regression analysis, increased eCAT volume was independently associated with all cardiac events. However, when maximum lumen narrowing was additionally considered in the model, increased eCAT volume was no longer predictive (Table 4).

**Table 3:** Univariate Cox proportional-hazard regression for predictors of all cardiac events.

Variable	HR	95% CI	P value
Age, years	0.96	0.91 to 1.01	0.12
>3 risk factors *	1.45	0.39 to 5.37	0.58
Presence of CAD	0.17	0.02 to 1.33	0.09
Hs-CRP, mg/l	1.11	0.96 to 1.28	0.15
Hs-TnT, pg/ml	1.0	0.90 to 1.10	0.92
Increases eCAT volume, ≥ 162.2 cm^3^*	**4.08**	1.28 to 12.97	**0.017**
Maximal lumen narrowing in CCTA, %	**1.05**	1.01 to 1.10	**0.009**

*Dichotomous variable; CAD indicates coronary artery disease; HR, hazard ratio; 95% CI, 95% confidence interval; hs-CRP, high-sensitive C-reactive protein; hs-TnT, high-sensitive Troponin T; CCTA, cardiac computed tomography angiography.

**Table 4:** Two models (A and B) of multivariable Cox proportional-hazard regression analysis for predictors of all cardiac events.

Variable	b-coefficient	HR	95% CI	P value
	**Model A**			
Age, years	-0.07	0.94	0.87 to 1.01	0.09
>3 risk factors *	-0.47	0.63	0.10 to 3.15	0.57
Presence of CAD	-1.68	0.19	0.20 to 1.53	0.12
Hs-CRP, mg/l)	0.08	1.08	0.90 to 1.30	0.40
Hs-TnT, pg/ml)	0.001	1.00	0.91 to 1.10	0.99
Increases eCAT volume, ≥ 162.2 cm^3^*	1.59	**4.89**	1.23 to 19.35	**0.02**
	**Model B**			
Age, years	-0.06	0.94	0.87 to 1.02	0.17
>3 risk factors*	-14.80	0.00	0.00 to 0.00	0.96
Presence of CAD	0.40	1.49	0.14 to 15.52	0.74
Hs-CRP, mg/l	0.24	1.28	0.87 to 1.87	0.21
Hs-TnT, pg/ml	-0.07	0.93	0.82 to 1.06	0.27
Increases eCAT volume, ≥ 162.2 cm^3^*	2.15	*8*.*60*	0.90 to 82.74	*0*.*06*
Maximal lumen narrowing in CCTA, %	0.09	**1.10**	1.02 to 1.18	**0.02**

*Dichotomous variable; CAD indicates coronary artery disease; HR, hazard ratio; 95% CI, 95% confidence interval; hs-CRP, high-sensitive C-reactive protein; hs-TnT, high-sensitive Troponin T; CCTA, cardiac computed tomography angiography.

Kaplan-Meier survival analysis demonstrated a prognostic value of elevated eCAT volume for cardiac events (HR = 4.1, chi-square = 6.7, p = 0.009, Fig 5B). Patients with elevated eCAT volume showed an increased annual event rate of 4.2% versus 1.0% for patients with lower eCAT volume. A series of hierarchical Cox proportional-hazards regression models demonstrated an incremental predictive value of elevated eCAT volume to presence of CAD and presence of plaque (S2A & S2B Fig). In this line, no reclassification improvement resulted for elevated eCAT volume (net reclassification improvement, NRI = 19.5%) compared to maximum luminal narrowing (NRI = 26.9%) and total plaque volume (NRI = 55.0%), when added to age, diabetes mellitus, hyperlipidemia and BMI.

### Observer agreement and variabilities and time-spent

The threshold-based eCAT volume assessment provided good intra-observer and inter-observer ICC of 0.9923 (95%CI 0.9785 to 0.9973) and 0.9996 (95%CI 0.9851 to 1.0000), respectively. Quantitative assessment required a mean interpretation time of 4.5±1.1min and 3.9±3.1 min per patient for eCAT volume and plaque characterization, respectively.

## Discussion

In the present study we demonstrate a significant association of elevated epicardial adipose tissue (eCAT) volume with increased coronary atherosclerotic plaque burden, hs-TnT and hs-CRP. ECAT volume provided incremental prognostic value to traditional risk factors, presence of coronary artery disease (CAD), hs-CRP and hs-TnT in patients with presumably stable CAD. These findings may indicate an additional potential paracrine impact of eCAT on coronary plaque vulnerability that is different from accepted molecular trigger of atherosclerosis inception and progression.

### ECAT and coronary plaque burden and composition

Among visceral fat, eCAT represents a unique sub-compartment first due to its close proximity to the heart muscle and the coronary arteries, and second due to its inflammatory activity [31]. In this context, paracrine and vasocrine effects of inflammatory cytokines from eCAT may promote atherogenesis and lead to elevated risk of adverse coronary events [11] (S3 Fig). Several investigations have described a significant association of eCAT volume with the presence of CAD and coronary plaque burden, which is in part explained by the strong link between eCAT and atherogenic risk factors [3, 5, 8, 11]. In this line, we demonstrated an independent association of eCAT volume with the presence and severity of CAD after the adjustment for age, diabetes mellitus, hyperlipidemia, body-mass-index (BMI), hs-CRP and hs-TnT. Using quantitative plaque assessment, we demonstrated an independent association of elevated eCAT volume with total plaque volume, total number of plaques and coronary lumen narrowing. Patients suffering from relevant CAD exhibited the highest eCAT volumes. In the past years several studies demonstrated a close association of eCAT volume with clinical parameters such as BMI and atherogenic risk factors [3, 12]. We detected an inverse correlation of eCAT volume with HDL-cholesterol, while serum levels of triglycerides were positively related to eCAT volume, which is in agreement with prior results [32].

The impact of atherogenic risk factors on plaque composition was assessed in several large-scale clinical studies [15, 17, 27]. Furthermore, a strong association of eCAT volume with non-calcified plaque components was previously reported [5, 8, 9, 22]. In the present study we demonstrated a significant BMI-independent correlation of elevated eCAT volume with total plaque volume, fraction of non-calcified plaque volume and total calcium score, which underscores the suggested association of eCAT volume with calcific and non-calcific plaque burden [3, 13].

### ECAT, biomarkers and cardiac outcomes

Results from basic and clinical research propose that a mismatch of several pro- and anti-inflammatory cytokines and mediators secreted from the eCAT may locally impact on atherogenesis in the underlying coronary arteries [31, 33, 34]. Our reported results demonstrate, that patients with augmented hs-CRP reveal higher eCAT volumes independent of BMI, which may be due to the pro-inflammatory endocrine activity of eCAT volume. Of interest, we also identified an association with small increases of hs-TnT, which is an established biomarker for myocardial micro-injury [17, 35]. As with other prior investigations, we identified a significant association of minor increases of cardiac troponin T with vulnerable plaque characteristic as assessed by CCTA in patients with presumably stable CAD, which is possibly caused by silent plaque rupture, micro-embolization and microvascular obstruction, which may precede the clinical manifestation of myocardial infarction [15, 17, 35, 36]. The present results affirm that hs-TnT correlates with coronary plaque burden as assessed by total plaque volume and calcium scoring. Recently, a report from the Heinz Nixdorf Recall Study reinforced the hypothesis that elevated eCAT volume drives disease progression predominantly in early stages of atherosclerosis [37]. In this line, our results give further evidence that eCAT volume is not only a bystander, but may be a key player for plaque progression and formation of vulnerable coronary lesions above and beyond the traditional mechanisms of plaque progression.

Several investigations have demonstrated that eCAT volume is associated with incident cardiovascular events [6, 13, 38]. In our study, patients with elevated eCAT volume exhibited an increased risk for future cardiac events. Using a series of hierarchical Cox proportional-hazards regression models we demonstrated an incremental value of elevated eCAT volume to age, atherogenic risk factors, presence of CAD, hs-CRP and hs-TnT for the prediction of all cardiac events. However, when maximum luminal narrowing was considered in the model, increased eCAT volume was no longer predictive. Therefore, our results contribute to an expanding body of evidence for the role of eCAT volume in destabilization of vulnerable lesions, resulting in a higher incidence of cardiovascular events.

### Limitations

The strength of our study is the unique complementary assessment of quantitative CCTA-based plaque characteristics and eCAT volume in conjunction with biomarkers for inflammation (hs-CRP) and myocardial micro-injury (hs-TnT). However, the major limitation of the presented study is the relatively small number of patients and cardiac endpoints. Second, no mechanistic data on paracrine or vasocrine inflammatory effects of eCAT on coronary plaque composition were assessed. Especially, the clinical significance of the weakly correlated eCAT volume with biomarkers, and its association with plaque burden and lumen narrowing needs to be investigated in future large-scale clinical trials to reinforce our findings. Finally, lipid serum assessments were accessible in only 55% of the study population.

### Conclusions

Epicardial adipose tissue (eCAT) volume is independently associated with atherosclerotic plaque burden and CAD severity as assessed by cardiac computed tomography angiography (CCTA) and hs-TnT as biomarker of myocardial micro-injury. Elevated eCAT volume may provide incremental predictive value for future cardiac events in patients at intermediate risk for coronary artery disease (CAD).

## Supporting Information

S1 Appendix (DOCX)

S1 Fig Linear regression analysis revealed a close correlation of epicardial adipose tissue (eCAT) volume with serum levels of high-density lipoprotein (HDL, r = -0.39, p = 0.001, 95% CI -0.58 to -0.15) and triglycerides (r = 0.36, p = 0.004, 95% CI 0.12 to 0.56), whereas no significant correlation appeared for total cholesterol (r = -0.19, p = 0.114, 95% CI -0.40 to 0.05), and low-density lipoprotein (LDL, r = -0.15, p = 0.25, 95% CI -0.39 to 0.11), respectively. ECAT indicates epicardial adipose tissue.
(TIFF)

S2 Fig Series of hierarchical Cox proportional-hazards models identifies an incremental predictive value for all cardiac events (non fatal myocardial infarction, cardiac death, late revascularization) for elevated eCAT (epicardial adipose tissue) volume (≥162.2 cm^3^). The fist model (**A**) includes ‘presence of CAD’, the second model (**B**) ‘presence of plaque’. DM, diabetes mellitus; BMI, body-mass-index; CAD, coronary artery disease; CCTA, cardiac computed tomography angiography; hs-CRP, high-sensitive C-reactive protein; hs-TnT, high-sensitive Troponin T.
(TIFF)

S3 Fig Schematic illustration of the potential role of epicardial adipocytes on coronary atherosclerosis, plaque destabilization and myocardial micronecrosis. IL-1β indicates interleukin 1β; IL-6, interleukin 6; MCP, macrophage chemoattractant protein; TNF-α, Tumor necrosis factor-α; hs-CRP, high-sensitive C-reactive protein; hs-TnT, high-sensitive Troponin T.
(TIFF)
