# Supplementary material for: Epicardial Adipose Tissue Is Associated with Plaque Burden and Composition and Provides Incremental Value for the Prediction of Cardiac Outcome. A Clinical Cardiac Computed Tomography Angiography Study
Source: PLoS One. 2016 May 17;11(5):e0155120. doi: 10.1371/journal.pone.0155120 (PMC4871366; doi:10.1371/journal.pone.0155120)
Supplement: S1 Appendix — (DOCX) [file pone.0155120.s001.docx]

**Online Appendix.**

**Inclusion and exclusion criteria.** The inclusion criterion was the scheduling for CCTA due to clinical indication according to the current guidelines ([1](#_ENREF_1)). Exclusion criteria were non-sinus rhythm or arrhythmia (n=2), acute coronary syndrome, cardiomyopathies, acute myocarditis, moderate or severe valvular disease, elevated serum creatinine (>1.5 mg/dl, n=1), history or signs of previous myocardial infarction or revascularization (n=11) and severe iodine allergy. Seven patients were excluded due to non-diagnostic images (n=3) or severe coronary calcifications (Agatston Score >800, n=4). One patient was excluded due to refusal for contrast material (n=1).

**Demographic and clinical characteristics.** Patient body weight, height and body mass index (BMI) and traditional CAD risk factors like 1.) advanced age (>65yrs.), 2.) male gender, 3.) arterial hypertension (blood pressure≥140/90 mmHg or antihypertensive therapy), 4.) hyperlipidemia (triglycerides≥190mg/dL or LDL-cholesterin≥160mg/dL or statin treatment), 5.) cigarette smoking (self-reported), 6.) diabetes mellitus (HbA1c>6.5% or antidiabetic treatment), 7.) family history of CAD, self-reported) and cardiac medications were recorded at the time of the CCTA (Table 1). Based on the sum of the atherogenic risk factors mentioned above (1.-7.) a score was built (range 0-7) and the Duke Clinical Score was calculated ([2](#_ENREF_2)).

**Coronary plaque volume and composition.** For each coronary artery segment the vessel lumen and wall were automatically registered, and after identification of each lesion the boundaries were manually edited by G.G. or G.K. if necessary. Subsequently, the semi-automatically identified plaques were marked. These findings were then evaluated by experienced observers (G.G. & G.K) in corresponding axial, cross-sectional multiplanar and longitudinal images in order to differentiate real findings from artifacts. Care was taken to correctly discriminate between iodinated blood (300 to 600 HU) and calcified plaque, and Gaussian algorithms were used to distinguish between components of low to intermediate attenuation (0 to 150 HU) and calcified plaque components with higher attenuation values ([3-5](#_ENREF_3)). This model separates components with different densities within the plaque using a Gaussian mixture model. Finally, plaque composition results from a linear combination of the resulting 1-3 Gaussians curves. For each lesion the following were assessed: plaque volume, coronary lumen narrowing, plaque composition and vascular remodeling. According to the volumetric calcium content, plaques were classified into non-calcified (calcium content <20%), partially calcified (calcium content between 20% to 80%) and calcified (calcium content >80%). Non-calcified and partially calcified plaques are expected to contain substantial amount of lipid cores or fibrotic tissues ([4](#_ENREF_4),[6](#_ENREF_6)) apart from calcified tissue. The non-calcified plaque volume for each individual lesion and each patient was obtained by summing the individual volumes of non-calcified or mixed plaques in all three coronary vessels.

**Luminal narrowing.** Coronary lumen narrowing (maximum diameter reduction) was analyzed using longitudinal reconstructions by dividing the minimal diameter in the diseased segment through the diameter in the adjacent proximal disease-free section. If stenosis was present at the ostium of a coronary artery or a vessel bifurcation point, the distal or the proximal reference vessel point were used, respectively. When multiple lesions were present in a segment, the most severe lesion was considered. The severity of luminal stenosis was divided into non-obstructive and obstructive lesions (<70% versus ≥70% stenosis) ([7](#_ENREF_7)). In addition, maximum of luminal stenosis was quantified in each patient.

**Biomarkers.** Blood samples were drawn from a peripheral vein 2 h before the CCTA scan. Samples were stored immediately at -80°C and were analyzed at a later time point. Thus, the clinicians did not know hs-TnT values and the management of our patients was therefore not influenced by hs-TnT values. Hs-TnT was measured using the new hsTnT quantitative electrochemiluminescence immunoassay (Cobas 411, Roche Diagnostics, Mannheim, Germany) as described previously ([8](#_ENREF_8)). Samples were analyzed in batches, using hs-TnT lots unaffected by a lot-to-lot variation issue (Lot numbers 164773 &167650), which was recently reported to cause a downshift of hs-TnT concentrations ([6](#_ENREF_6)). The assay is specific for Troponin T without relevant interferences and has an analytic range of 3–10000 ng/L (limit of the blank/LOB 3 ng/L, limit of quantification/LOQ 14 ng/L). A concentration of 14 ng/L has been identified as the 99th percentile of a healthy reference population with a CV of <10%. Routine laboratory parameter measurements including hs-CRP and creatinine were performed at the core laboratory of our hospital.

**References**

1. Task Force M, Montalescot G, Sechtem U et al. 2013 ESC guidelines on the management of stable coronary artery disease: the Task Force on the management of stable coronary artery disease of the European Society of Cardiology. European heart journal 2013;34:2949-3003.

2. Pryor DB, Shaw L, McCants CB et al. Value of the history and physical in identifying patients at increased risk for coronary artery disease. Ann Intern Med 1993;118:81-90.

3. Korosoglou G, Mueller D, Lehrke S et al. Quantitative assessment of stenosis severity and atherosclerotic plaque composition using 256-slice computed tomography. European radiology 2010;20:1841-50.

4. Motoyama S, Sarai M, Harigaya H et al. Computed tomographic angiography characteristics of atherosclerotic plaques subsequently resulting in acute coronary syndrome. Journal of the American College of Cardiology 2009;54:49-57.

5. Korosoglou G, Ivanc TB, Mueller DK, Katus HA. Assessment of atherosclerotic plaque composition using 256-slice CT and association with biochemical markers. MEDICAMUNDI 2011;55.

6. Korosoglou G, Lehrke S, Mueller D et al. Determinants of troponin release in patients with stable coronary artery disease: insights from CT angiography characteristics of atherosclerotic plaque. Heart 2011;97:823-31.

7. Hadamitzky M, Distler R, Meyer T et al. Prognostic value of coronary computed tomographic angiography in comparison with calcium scoring and clinical risk scores. Circulation Cardiovascular imaging 2011;4:16-23.

8. Giannitsis E, Kurz K, Hallermayer K, Jarausch J, Jaffe AS, Katus HA. Analytical validation of a high-sensitivity cardiac troponin T assay. Clinical chemistry 2010;56:254-61.
