# Supplementary figures and images for: Epicardial Adipose Tissue Is Associated with Plaque Burden and Composition and Provides Incremental Value for the Prediction of Cardiac Outcome. A Clinical Cardiac Computed Tomography Angiography Study
Source: PLoS One. 2016 May 17;11(5):e0155120. doi: 10.1371/journal.pone.0155120 (PMC4871366; doi:10.1371/journal.pone.0155120)

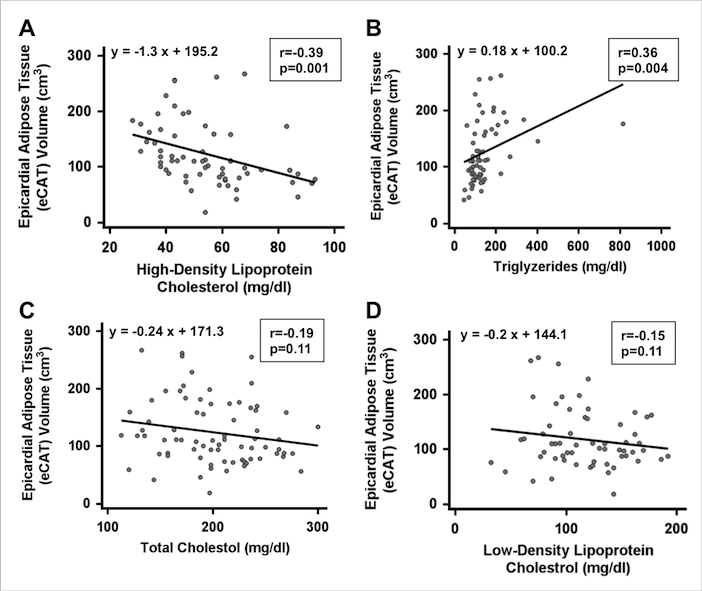

Supplement: S1 Fig — ECAT indicates epicardial adipose tissue. (TIFF) [file pone.0155120.s002.tiff]

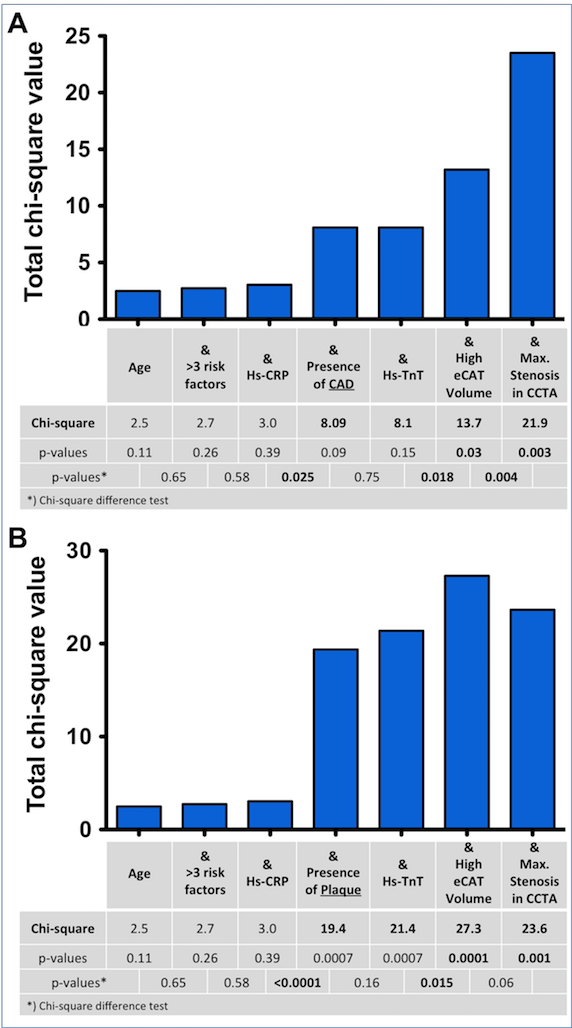

Supplement: S2 Fig — The fist model (A) includes ‘presence of CAD’, the second model (B) ‘presence of plaque’. DM, diabetes mellitus; BMI, body-mass-index; CAD, coronary artery disease; CCTA, cardiac computed tomography angiography; hs-CRP, high-sensitive C-reactive protein; hs-TnT, high-sensitive Troponin T. (TIFF) [file pone.0155120.s003.tiff]

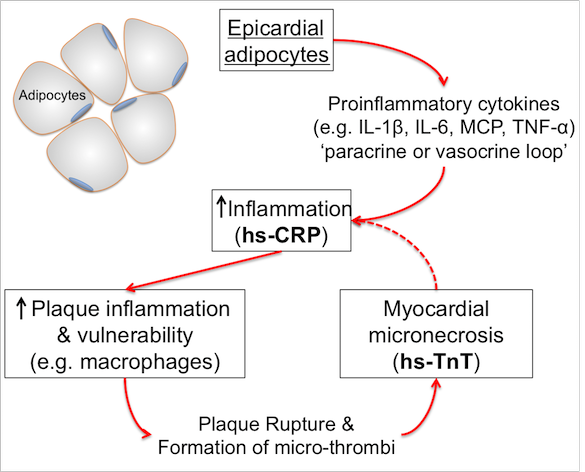

Supplement: S3 Fig — IL-1β indicates interleukin 1β; IL-6, interleukin 6; MCP, macrophage chemoattractant protein; TNF-α, Tumor necrosis factor-α; hs-CRP, high-sensitive C-reactive protein; hs-TnT, high-sensitive Troponin T. (TIFF) [file pone.0155120.s004.tiff]
